# Scalable Manufacturing Method for Model Protein-Loaded PLGA Nanoparticles: Biocompatibility, Trafficking and Release Properties

**DOI:** 10.3390/pharmaceutics17010087

**Published:** 2025-01-10

**Authors:** Selin Akpinar Adscheid, Marta Rojas-Rodríguez, Salma M. Abdel-Hafez, Francesco S. Pavone, Marc Schneider, Akif E. Türeli, Martino Calamai, Nazende Günday-Türeli

**Affiliations:** 1 MyBiotech GmbH, Industriestraße 1B, 66802 Überherrn, Germany; seak00004@uni-saarland.de (S.A.A.); e.tuereli@mybiotech.de (A.E.T.); 2 Department of Pharmacy, Biopharmaceutics and Pharmaceutical Technology, PharmaScienceHub, Saarland University, Campus C4 1, 66123 Saarbrücken, Germany; salma.abdelhafez@pharma.asu.edu.eg; 3 European Laboratory for Non-Linear Spectroscopy, via Nello Carrara 1, 50019 Sesto Fiorentino, Italy; rojas@lens.unifi.it (M.R.-R.); pavone@lens.unifi.it (F.S.P.); calamai@lens.unifi.it (M.C.); 4 Department of Pharmaceutics and Industrial Pharmacy, Faculty of Pharmacy, Ain Shams University, Cairo 11566, Egypt; 5 Department of Physics and Astronomy, University of Florence, Via G. Sansone 1, 50019 Sesto Fiorentino, Italy; 6 National Institute of Optics-National Research Council (CNR-INO), 50125 Sesto Fiorentino, Italy

**Keywords:** confocal imaging, drug delivery, drug release, drug trafficking, intranasal drug delivery, nanoparticles, nanoparticle manufacturing, polymeric nanoparticles, scalability

## Abstract

**Background and Objectives**: Drug delivery systems (DDSs) offer efficient treatment solutions to challenging diseases such as central nervous system (CNS) diseases by bypassing biological barriers such as the blood–brain barrier (BBB). Among DDSs, polymeric nanoparticles (NPs), particularly poly(lactic-co-glycolic acid) (PLGA) NPs, hold an outstanding position due to their biocompatible and biodegradable qualities. Despite their potential, the translation of PLGA NPs from laboratory-scale production to clinical applications remains a significant challenge. This study aims to address these limitations by developing scalable PLGA NPs and evaluating their potential biological applications. **Methods**: We prepared blank and model-protein-loaded (albumin–FITC and wheat germ agglutinin-488 (WGA-488)) fluorescent PLGA NPs using the traditional double-emulsion method combined with the micro-spray-reactor system, a novel approach that enables fine particle production enabling scale-up applications. We tested the biocompatibility of the NPs in living RPMI 2650 and neuroblastoma cell lines, as well as their trafficking and uptake. Release kinetics of the encapsulated proteins were investigated through confocal microscopy and in vitro release studies, providing insights into the stability and functionality of the released proteins. **Results**: The formulation demonstrated sustained and prolonged protein release profiles. Importantly, cellular uptake studies revealed that the NPs were not internalized. Furthermore, encapsulated WGA-488 protein retained its functional activity after release, validating the integrity of the encapsulation and release processes. **Conclusions**: The proof-of-concept study on NP manufacturing and an innovative drug trafficking and release approach can bring new perspectives on scalable preparations of PLGA NPs and their biological applications.

## 1. Introduction

Drug delivery systems (DDSs) play a vital role in treating a wide range of diseases by enhancing the stability, efficacy, and safety of therapeutic agents [1]. Among this wide range of diseases, treating the central nervous system (CNS) remains challenging due to the presence of the blood–brain barrier (BBB), which limits efficient drug delivery to the brain [2]. Although it is challenging, DDSs have already shown promising results in bypassing different biological barriers as well as promising results in preclinical and clinical studies regarding challenges such as treating CNS diseases [3]. Among different classes of DDS, polymeric nanoparticles (NPs) stand out due to their highly tunable and controllable properties [4,5]. Among polymeric NPs, poly(lactic-co-glycolic) acid (PLGA) NPs are an essential class, as PLGA is known to be biocompatible, biodegradable, and approved by the US Food and Drug Administration (FDA) for therapeutic use [6]. Although it is approved by regulatory agencies, there are still no PLGA-NP-based therapeutics available on the market [7].

From a manufacturing point of view, traditional small-scale laboratory manufacturing of polymeric NPs often suffers from batch-to-batch variability and lack of sufficient control in fine particle production [8]. Thus, one of the challenges in the transition from benchside to bedside of developing polymeric NPs is limited scale-up options. Although there are efforts on the scalability of DDSs [9,10,11,12], there is still a need for different manufacturing platforms to maximize the transition from lab-scale research to market applicability.

Therefore, this study introduces a unique manufacturing platform for preparing model-substance-loaded PLGA NPs by a modified emulsification/solvent evaporation (double-emulsion) method. The manufacturing technology, the micro-spray-reactor (MSR) system, is a bottom-up approach that involves the controlled mixing of solvent and non-solvent phases by spraying through a nozzle. This controlled mixing enables the interaction in each droplet and provides control over the fine particle productions. Moreover, the system offers interchangeable geometry and adjustable distance, enabling various methods such as precipitation and coating. To facilitate high encapsulation efficiency while still maximizing scale-up opportunities, we combined the MSR system with the traditional high-pressure homogenization method to produce PLGA NPs as double emulsions. The double-emulsion method offers high encapsulation efficiency for hydrophilic molecules [13,14,15].

In this method, a polymer (PLGA) is dissolved in a solvent, and hydrophilic drug substances are dissolved in an aqueous phase where both phases are emulsified with a high-shear external force to prepare the primary emulsion. The resulting primary emulsion is added into another aqueous phase and emulsified by the high-shear external force to form the double emulsion. At the end of the process, the resulting double emulsion is stirred to evaporate the solvent and solidify the polymer to form the NPs [16]. Applying the double-emulsion method to the MSR system enabled us to conduct proof-of-concept studies on the encapsulation of large hydrophilic molecules with the possibility of scale-up options. Using the MSR system in the second emulsification step in the double-emulsion method, we tuned the particle size of PLGA NPs to approximately the desired range. We enabled fine particle production without using a high-shear force homogenizer in the second emulsification step, where the larger volumes of the phases are generally used compared to the first emulsification step to prepare the primary emulsion. This effort can be considered a step further for easier scalability of the double-emulsion technique.

To test the compatibility of PLGA NPs manufactured by the MSR system for possible biological applications such as N2B delivery to treat CNS diseases, we prepared fluorescent PLGA NPs. As a large hydrophilic model substance, we chose fluorescent bovine serum albumin (BSA) and loaded it into the fluorescent PLGA NPs. As crossing the cell barrier rather than internalization is the target for applications such as N2B delivery, we assessed their biocompatibility in different cell lines, evaluated their uptake and trafficking, and determined the release kinetics of the encapsulated fluorescent albumin. Furthermore, we loaded the PLGA NPs with wheat germ agglutinin-488 (WGA-488), a cell membrane marker, to test the functionality of the drug substances after encapsulation and release.

## 2. Materials and Methods

### 2.1. Preparation of the Rhod-PLGA

PLGA Resomer RG 503 H (molecular weight (Mw): 24,000–38,000 g/mol, Evonik Industries, Darmstadt, Germany) was covalently labeled with Rhodamine B (rhod) (Sigma-Aldrich, Steinheim, Germany) based on the method described by Lababidi et al. [17]. In brief, PLGA (1 molar equivalent (eq), 1 g) and 4-(Dimethylamino)pyridine (DMAP) (Sigma-Aldrich, Steinheim, Germany) (0.1 eq, 0.4 mg) were dissolved in dry dichloromethane (DCM) (15 mL). Rhodamine B (1.1 eq, 17 mg) and N,N′-dicyclohexylcarbodiimide (DCC) (Sigma-Aldrich, Steinheim, Germany) (1.5 eq, 10 mg) were dissolved in dry DCM (Thermo Fisher Scientific, Darmstadt, Germany) (15 mL). The Rhodamine B/DCC solution was added to the PLGA/DMAP solution, followed by stirring for 24 h at room temperature. After 24 h, the reaction was quenched by adding MilliQ water (1 mL). DCM was eliminated using rotary evaporation at 50 °C under atmospheric pressure. The yielded Rhod-PLGA was dissolved in acetone (20 mL) using an ultrasonic bath and the solution was transferred to centrifugation tubes. The Rhod-PLGA was precipitated by adding an equal volume of ethanol (HPLC-grade) (Fisher Scientific, Schwerte, Germany), followed by centrifugation at 20,000× *g*, for 20 min, at 20 °C (Multifuge X1R, Thermo Fisher Scientific, Osterode am Harz, Germany) and discarding the supernatant. The product was redissolved in acetone (HPLC-grade) (Fisher Scientific, Schwerte, Germany) and washed 5 additional times (or until a clear supernatant was observed) using the same method. The final pellet obtained after pooling was sonicated for 20 min in a cooled ultrasonic bath, frozen at −80 °C, and lyophilized (Alpha 3-4 LSCbasic, Christ, Osterode am Harz, Germany) for 48 h. The product yield was approximately 88%.

### 2.2. Preparation of the Albumin-Loaded and Blank Rhod-PLGA NPs

**Figure 1:**
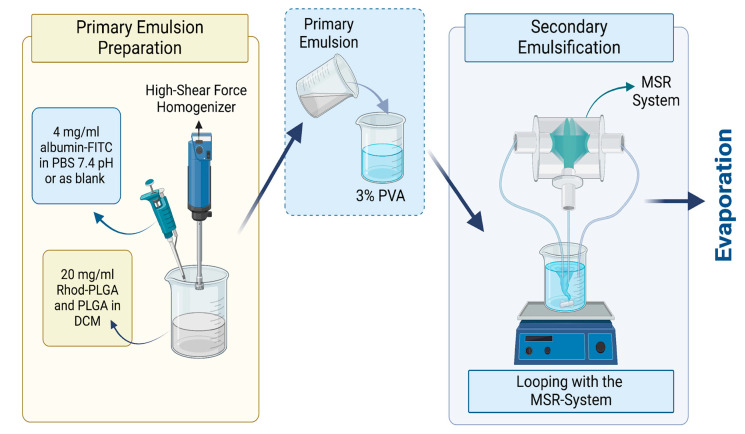
Preparation of the albumin–FITC-loaded Rhod-PLGA NPs. Created with Biorender.com.

Albumin–FITC-loaded Rhod-PLGA NPs were prepared by the modified emulsification/solvent evaporation method combined with the reactor system. The primary emulsion was prepared by adding 4 mg/mL albumin–fluorescein isothiocyanate (FITC) (bovine protein) (Sigma-Aldrich, Darmstadt, Germany) in phosphate-buffered saline (PBS) at 7.4 pH into a 20 mg/mL mixture of Rhod-PLGA and PLGA Resomer 502H (50:50 acid terminated; Mw: 7000–17,000, Evonik, Darmstadt, Germany) with a ratio of 1:20. The mixture was then dissolved in DCM (ACS Reagent, ISO, ≥99.9% (GC) grade, Merck KGaA, Darmstadt, Germany) with a phase ratio of 1:10. The emulsification was performed by an Ultra-Turrax^®^, a high shear force homogenizer, at 13,000 rpm for 60 s. Then, the primary emulsion was added into the 30 mg/mL Poly (vinyl alcohol) (PVA) (Mowiol^®^ 4-98, Mw ~27,000, Sigma–Aldrich, Munich, Germany) with a 1:5 ratio, and continuous looping through a 300 µm reactor was performed for 15 min with a flow rate of 200 mL/min in each pump (Figure 1). Finally, the PLGA double emulsion was stirred overnight to remove the organic solvent and form the PLGA NPs. For the MTT assay, instead of albumin–FITC, non-fluorescent albumin from Sigma Aldrich (Darmstadt, Germany) and non-fluorescent PLGA from Evonik (Darmstadt, Germany) were used.

### 2.3. Determination of the Encapsulation Efficiency for the Albumin–FITC-Loaded Rhod-PLGA NPs

To determine the concentration of the albumin–FITC in NPs, the sample was diluted in 1M NaOH (1:1) and centrifuged at 24,610 rcf for 10 min. The solution was read at the calibration curve by a microplate reader with a filter of 485 nm excitation and 590 nm emission wavelengths. To quantify the free albumin–FITC concentration, the sample was centrifuged at 24,610 rcf for 60 min. The supernatant was read at the same excitation and emission wavelengths to quantify the free albumin–FITC concentration. The percentage of encapsulated albumin–FITC concentration was calculated by subtracting the free albumin–FITC concentration from the total albumin–FITC concentration, which was divided by the total albumin–FITC concentration, multiplied by %.

### 2.4. Preparation of the WGA-488-Loaded PLGA NPs by the Bench-Top (Emulsification/Solvent Evaporation) Method

**Figure 2:**
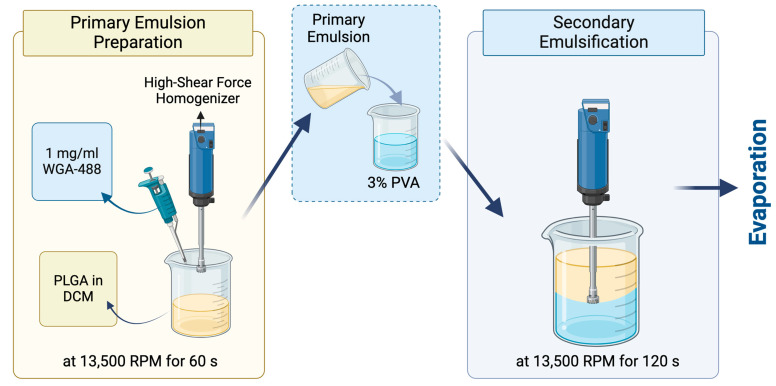
Preparation of the WGA-488-loaded PLGA NPs. Created with Biorender.com.

WGA-488-loaded PLGA NPs were prepared by the traditional bench-top (emulsification/solvent evaporation method) (Figure 2). The primary emulsion was prepared by adding 1 mg/mL wheat germ agglutinin (WGA) Alexa Fluor 488 (Invitrogen™, Waltham, MA, USA) in PBS at 7.4 pH into 20 mg/mL of PLGA Resomer 502H (50:50 acid terminated, Mw: 7000–17,000, Evonik, Darmstadt, Germany) dissolved in DCM with a phase ratio of 1:10. The emulsification was performed by the Ultra-Turrax^®^, a high-shear-force homogenizer, at 13,500 rpm for 60 s. Then, the primary emulsion was added into the 30 mg/mL PVA solution (Mowiol^®^ 4-98, Mw: ~27,000, Sigma–Aldrich, Munich, Germany) with a 1:5 ratio. The second emulsification was performed again with the Ultra-Turrax^®^ at the same rpm for 120 s. Finally, the PLGA double emulsion was stirred overnight to remove the organic solvent and form the PLGA NPs.

### 2.5. Purification of the PLGA NPs by Cross-Flow Filtration

Purification was performed by cross-flow filtration (CFF) with 300 kDa MicroKros© mPES (modified polyethersulfone) hollow fiber membranes (Repligen, Waltham, MA, USA) with a surface area of 235 cm^2^ to be sure to purify the sample from the free PLGA, surfactant, and albumin.

### 2.6. Lyophilization of NPs

For lyophilization, 50 mg/mL of d-Mannitol (Sigma-Aldrich, Darmstadt, Germany) was dissolved in nanosuspension and samples were introduced into the lyophilization program with a volume of 2 mL for each vial. To lyophilize the samples, a freezing step was performed at −70 °C for 7 h at 1000 mbar, followed by a two-step-long main drying at −40 °C for 6 h at 0.5 mbar, and at the same temperature at 0.120 mbar was maintained for 19 h. After the main drying, the secondary drying was performed for 20 h at 30 °C, at 0.050 mbar.

### 2.7. Particle Size Measurement

The colloidal characteristics of the prepared NPs were characterized by a Zetasizer (NanoZS90 from Malvern Instruments, Malvern, UK) using dynamic light scattering (DLS). The zeta average particle diameter, polydispersity index (PDI), zeta potential, and intensity distribution were checked by diluting the optimal samples with distilled water. The zeta average particle diameters were analyzed at a scattering angle of 90° at 25 °C.

### 2.8. Evaluating In Vitro Cumulative Release Profile

The cumulative release studies of albumin–FITC from albumin–FITC-loaded PLGA NPs were performed in PBS at pH 7.4 (n = 3). The lyophilized samples were resuspended in 5 mL PBS at pH 7.4 and stirred at 120 rpm. A 0.5 mL sample was collected and filtered through a syringe filter with 0.02 µm pore size (Whatman^®^, Anotop^®^, Maidstone, UK) to make sure that the NPs were retained and only free FITC–albumin was measured in the filtered solution. For this, a fine syringe membrane with a pore size of 0.02 µm was used to ensure the successful separation of the NPs and undissolved albumin from the released macromolecules. This approach aimed at avoiding any misleading high readings in analytical measurements during the release studies [18]. The sampling was performed at time intervals of 1 h, 2 h, 4 h, 24 h, and 48 h. The fluorescence intensity at each time interval was measured with a fluorometer with an excitation wavelength of 485 nm and emission wavelength of 590 nm.

### 2.9. RPMI 2650 Cell Culture

Nasal septum squamous carcinoma cells (RPMI-2650) (Leibniz Institute DSMZ (German Collection of Microorganisms and Cell Cultures GmbH), Braunschweig, Germany) were cultured in Eagle‘s Minimum Essential Medium (EMEM) (Sigma-Aldrich, Steinheim, Germany) supplemented with 10% fetal bovine serum (FBS) (Thermo Fisher Scientific, Darmstadt, Germany). The cells were subcultured once a week and the media were replaced with fresh media every other day. The cultures were maintained at 37 °C in a humidified atmosphere containing 5% CO_2_ (BD 260 Standard Incubator, Binder, Tuttlingen, Germany). The cells were used between passages 3 and 10.

### 2.10. Cell Viability MTT Assay

RPMI-2650 cells were seeded (150,000 cells/well) into 96-well plates and grown for 24 h. Afterwards, the cells were washed twice with Hank’s Balanced Salt Solution (HBSS) (Thermo Fisher Scientific, Darmstadt, Germany) buffer, and non-fluorescent albumin-loaded PLGA NPs suspended in HBSS buffer were added at different concentrations (50, 100, 200, 300, 400 and 500 μg/mL). The samples were tested against negative (HBSS buffer) and positive (2% Triton-X) controls. After 4 h incubation with the formulations and controls, the cells were washed twice with HBSS and incubated with thiazolyl blue tetrazolium bromide (MTT reagent) (Sigma-Aldrich, Steinheim, Germany) for 4 h, followed by aspiration and incubation with DMSO for 20 min. The absorbance of formazan formed by living cells was measured at 550 nm and cell viability was calculated. All experiments were performed in triplicates. The particle size of the NPs was checked in cell culture medium to ensure stability.

### 2.11. Neuroblastoma Cell Culture

Human SH-SY5Y neuroblastoma cells (American Type Culture Collection, Manassas, VA, USA) were cultured in Dulbecco’s Modified Eagle’s Medium (DMEM, Thermo Fisher Scientific, Waltham, MA, USA) supplemented with 1% penicillin/streptomycin solution and 10% fetal bovine serum (FBS). Cell cultures were incubated at 37 °C and 5% CO_2_ in a humidified atmosphere. The cultures were grown until reaching 90% confluency.

### 2.12. Live Cell Imaging for Localization and Release Studies with Neuroblastoma Cells and Confocal Microscopy Analysis

SH-SY5Y cells were plated on 18 mm glass coverslips in 12-well plates at 100,000 cells per well. Twenty-four hours after plating, cells were incubated with 100 μg/mL of NPs. The particle size of the particles was checked in cell culture medium to ensure the particles’ stability.

For the localization experiments, cells were incubated for 4 h with 100 μg/mL of blank Rhod-PLGA NPs in the regular medium and, subsequently, with 5 μg/mL WGA-488 for 30 min to specifically label the plasma membrane. Ten minutes before the time finished, cells were incubated with Hoechst 33342 dye at a concentration of 10 μg/mL in order to stain the nuclei of the cells. After the incubation, cells were washed with PBS and the medium was replaced with Leibovitz’s L-15 (Thermo Fisher Scientific, Waltham, MA, USA) before imaging, a medium designed for supporting cell growth in the absence of CO_2_ equilibration.

For the time-course experiments, cells were first incubated for ten minutes with Hoechst, then washed with PBS and mounted on a custom chamber for imaging, and the incubation medium was replaced with Leibovitz’s L-15. Subsequently the Rhod-PLGA loaded with albumin–FITC NPs or WGA-488-loaded PLGA NPs were added and followed for 60 min using a laser scanning confocal microscope (LSCM). For the 4 h and 24 h timepoints, cells were incubated with 100 μg/mL of NPs in the regular medium. Ten minutes before the time finished, cells were incubated with Hoechst. After the incubation, cells were washed with PBS and the medium was replaced with Leibovitz’s L-15 before imaging.

The analysis of blank Rhod-PLGA NPs and Rhod-PLGA loaded with albumin–FITC NPs was performed after excitation at 561, 488, and 405 nm. The analysis of PLGA NPs loaded with WGA-488 was performed after excitation at 488 and 405 nm. A Nikon C2 LSCM and a Plan Apo λ 100X 1.45 NA oil immersion objective were used. For each sample, optical sections at median planes of the cells were taken (1024 × 1024 pixels) using sub-saturation settings, and all of them were kept constant for each analysis (laser power, detector gain, and pinhole diameter). Images were processed and analyzed with the Fiji software (ImageJ 2.16.0) and Origin (Pro) (“Version 2022b”, OriginLab Corporation, Northampton, MA, USA).

To characterize the co-localization degree between the Rhod-PLGA NPS and the plasma membrane, as well as the cargo albumin–FITC and the Rhod-PLGA NPs, the JACoP plugin [19] of Fiji software was used, specifically to calculate Pearson’s correlation coefficient [20], which estimates the covariance of signal intensities between two images by analyzing them on a pixel-by-pixel basis, and Manders’ overlap coefficient [21], which measures the fraction of pixels in image 1 that overlap with those in image 2, and vice versa. The data were analyzed with Origin (Pro).

## 3. Results and Discussion

### 3.1. Preparation and Characterization of NPs

To quantify the amount of encapsulated albumin in the PLGA NPs and visualize both the PLGA particles and the loaded albumin with confocal microscopy during the incubation with living cells, both albumin and the PLGA were tagged with fluorescent dyes, FITC and Rhodamine B, respectively. The FITC and the Rhodamine B dyes were chosen according to their distinct excitation and emission profiles. The albumin–FITC-loaded Rhod-PLGA NPs were prepared by combining the traditional homogenization method with the MSR system. The resulting fluorescent NPs exhibited a mean particle size of 241 nm and a PDI value < 0.2, indicating narrow particle size distribution in NP formation. The zeta potential of the NPs was measured as −30 mV after encapsulation. After lyophilization, the mean particle size slightly decreased to 220 nm. A comparison with blank Rhod-PLGA NPs, which had a mean particle size of 222 nm and a zeta potential of −32 mV, revealed that while the encapsulation of albumin–FITC led to a slight increase in particle size, the zeta potential values remained similar. This suggests that albumin–FITC was encapsulated into the NPs and did not alter the surface charge. After the lyophilization, the mean particle size of Rhod-PLGA NPs slightly decreased to 206 nm.

The following assay results showed an encapsulation efficiency as high as 84% and drug loading capacity of 2% (*w*/*w*). Fluorescent NPs were purified by the CFF process using a 300 kDa molecular weight cut-off (MWCO) membrane to sufficiently remove excess amounts of polymer, surfactant, and the free, non-encapsulated albumin–FITC. The hollow fiber membrane with 300 kDa MWCO was chosen as it provides optimal balance between the NP retention and removal of the smaller molecules such as PVA, PLGA, and encapsulated substances. Moreover, Dalwadi et al. previously showed that MWCO with a 300 kDa membrane size sufficiently removed the excess PVA in large batches and demonstrated better performance than ultracentrifugation, further supporting the choice of this membrane cut-off [22].

After lyophilization, the mean particle size slightly decreased to 221 nm. Overall, the lyophilization processes yielded a homogeneous lyophilization cake. To test NPs’ stability in the cell culture medium for further studies, the particle size measurements in cell culture medium EMEM as well as in water were compared. During the medium stability test for Rhod-PLGA NPs, the mean particle size in EMEM was measured as 188 nm, while in water, this value was 199 nm. For albumin–FITC-loaded PLGA NPs, the mean particle size and PDI values in EMEM were measured as 167 nm and 0.24, while in water, 196 nm and 0.02 were determined. The results showed that despite slight changes in the particle sizes and PDI values, the NPs remained stable in the medium. In order to characterize the co-localization of albumin–FITC with Rhod-PLGA NPs, we calculated Pearson’s and Manders’ coefficients from confocal images. We obtained a Pearson coefficient value of 0.77 ± 0.016 and Manders’ M1 = 0.84 ± 0.049 and M2 = 0.74 ± 0.019, where M1 is related to the degree of overlapping of albumin–FITC with the Rhod-PLGA NPs, and M2 is the overlapping of Rhod-PLGA NPs to albumin–FITC. These values, close to 1, indicate a high degree of co-localization.

### 3.2. Preparation and Characterization of WGA-488-Loaded PLGA NPs

The WGA-488-loaded PLGA NPs were prepared to test that the structural integrity of the protein cargo and its recognition and binding features were retained after encapsulation by the emulsification/solvent procedure. WGA specifically binds N-acetyl-D-glucosamine and sialic acid, and its fluorescent form is commonly used to label the plasma membrane [23]. The DLS measurements highlighted that the prepared WGA-488-loaded PLGA NPs were narrowly distributed with a mean particle size of 441 nm, zeta potential value of −52 mV, and PDI value < 0.2. After lyophilization, the mean particle size slightly decreased to 425 nm. Overall, the lyophilization processes yielded a homogeneous lyophilization cake. The particle sizes were also measured in cell culture medium to ensure stability. During stability-in-medium studies, the mean particle size in cell culture medium was measured as 395 nm, and that for dilution in water was measured as 412 nm, indicating that the particles were stable in the culture medium.

### 3.3. Biocompatibility of Albumin-Loaded PLGA NPs

**Figure 3:**
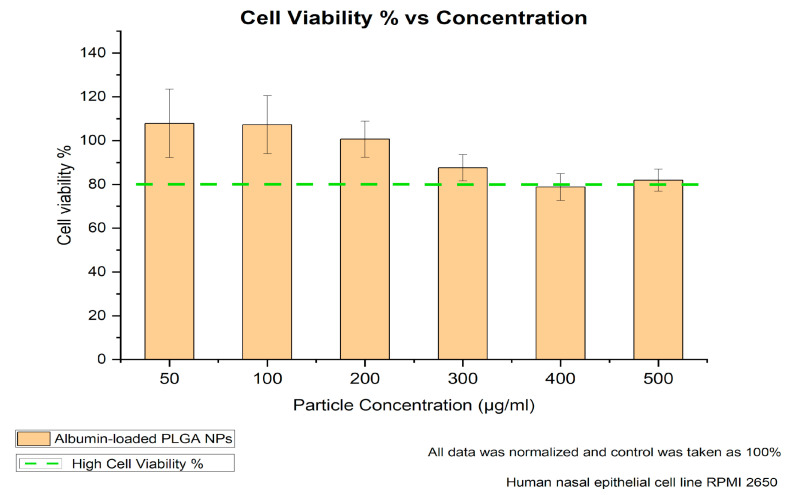
MTT assay results after 4 h of incubation for varying concentrations.

The viability of the treated non-fluorescent albumin-loaded PLGA-NPs and untreated cells of the nasal mucosa was investigated using the appropriate assay. In the literature, a study by Katsikari et al. showed significantly reduced viability above a 500 µg/mL particle concentration for albumin–FITC-loaded PLGA NPs prepared with the traditional double-emulsion method [24]. Therefore, our 4 h cell viability study further focused on the 50 to 500 µg/mL particle concentration range to evaluate the cellular response over a time span meaningful for intranasal administration conditions. Figure 3 shows that the RPMI-2650 nasal cells exhibited high cell viability over the studied particle concentrations, indicating their convenience for nasal application. As also explained by Katsikari et al., a further increase in particle concentration could negatively affect cell viability due to several factors, including particle size and surface properties, in addition to polymer degradation products’ interaction with the cell functions.

### 3.4. Rhod-PLGA NPs Are Not Internalized in the Cytoplasm

**Figure 4:**
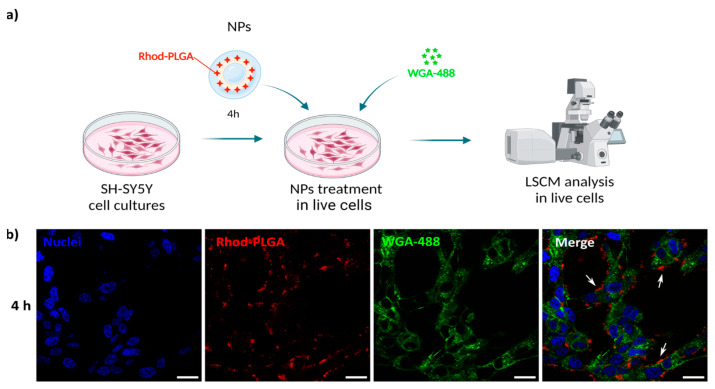
Rhod-PLGA NP localization. (**a**) Live cell experiment workflow. Created with Biorender.com. (**b**) Confocal scanning microscope images of neuroblastoma SH-SY5Y cells incubated for 4 h with 100 μg/mL of NPs and then with 5 μg/mL WGA-488 for 30 min to specifically label the plasma membrane. Red, Rhod-PLGA; green, WGA-488; blue, Hoechst. White arrows show the NPs’ position. Scale bar: 20 μm.

To follow the potential uptake of the NPs, human SH-SY5Y neuroblastoma cells were incubated with 100 μg/mL blank Rhod-PLGA NPs for 4 h and subsequently with WGA-488 to label the plasma membrane. The media were then washed and replaced before image acquisition (Figure 4a). The NPs were present only in the extracellular space, juxtaposed to the cells, without any noticeable internalization taking place (Figure 4b), not even after 24 h (Figure S1). This result is further corroborated by analyzing the degree of co-localization between the Rhod-PLGA NPs and the staining with WGA-488. The obtained Pearson’s coefficient value of 0.14 ± 0.008 and Manders’ M1 = 0.032 ± 0.025 and M2 = 0.16 ± 0.069 (where M1 is related to the degree of overlapping of WGA-488 staining with the Rhod-PLGA NPs, and M2 the overlapping of Rhod-PLGA NPs to WGA-488) indicate a very low degree of co-localization, supporting the lack of internalization of the Rhod-PLGA NPs.

The fact that these NPs were not internalized by the cells is a desirable characteristic for the possible N2B delivery application. If NPs can become internalized initially by the cells of the olfactory epithelium, the efficient targeting of distant regions of the CNS would become challenging, especially for low doses of NPs. In some neurodegenerative disorders, extracellular release is also a desired requirement (for instance, antibodies against Aβ oligomers for Alzheimer’s disease, or against anti-remyelinating receptors in the case of multiple sclerosis [1,25]). In addition, the lack of internalization can reduce NPs’ toxicity and off-target effects, both things of crucial importance when delivering drugs to sensitive regions like the brain, leading to a safer and more focused delivery system for treating neurological disorders.

### 3.5. In Vitro Cumulative Release Profile of PLGA NPs

**Figure 5:**
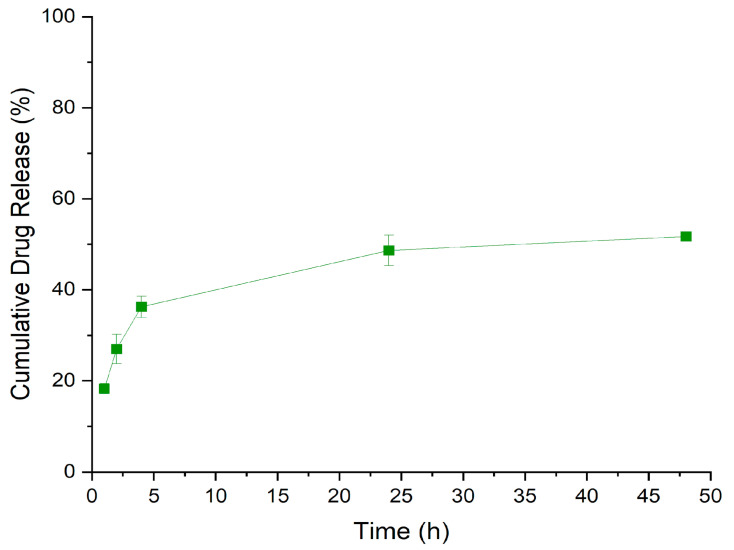
Cumulative release profile from the albumin–FITC-loaded PLGA NPs.

The cumulative drug release studies showed that the percent of drug released increased over the first 4 h, followed by a slight increase up to 24 h. Between 24 h and 48 h, the cumulative release only slightly further increased until approx. 50% released drug, indicating that the release of albumin–FITC from the PLGA NPs continues beyond this time frame (Figure 5).

### 3.6. Release from Albumin–FITC-Loaded Rhod-PLGA NPs

**Figure 6:**
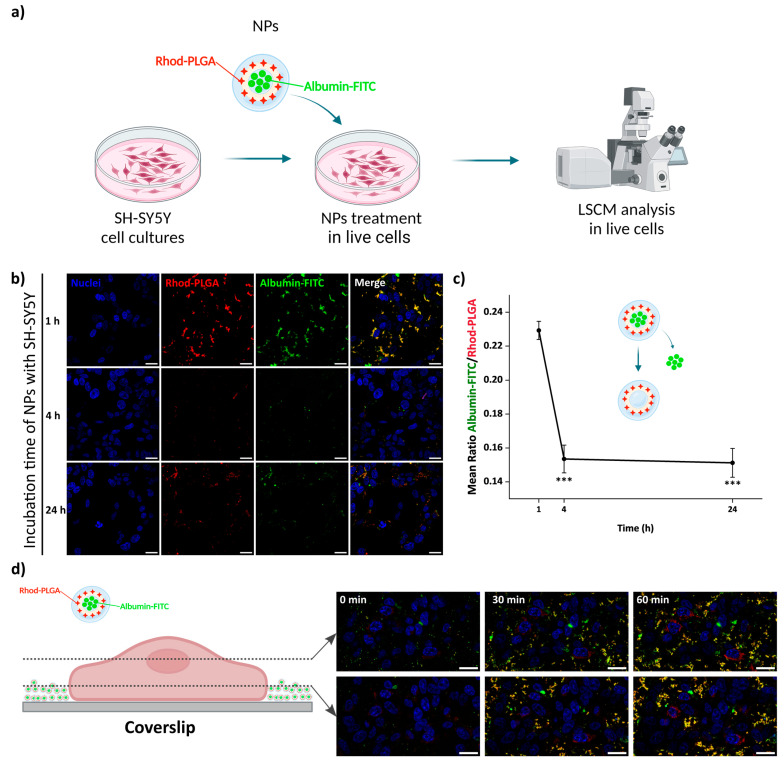
Albumin–FITC-loaded Rhod-PLGA NPs’ localization and release. (**a**) Live cell experiment workflow. Created with Biorender.com. (**b**) Confocal scanning microscope images of neuroblastoma SH-SY5Y cells incubated for different amounts of time with 100 μg/mL of NPs. (**c**) Quantification of the ratio between the albumin–FITC (cargo—green channel) to Rhod-PLGA (NPs—red channel) over 24 h. Error bars: SE; asterisks indicate significant differences between the measurements at 1 h using Kruskal–Wallis ANOVA test followed by Dunn’s multiple comparison post hoc test (*** *p* < 0.001). (**d**) Confocal images of SH-SY5Y cells incubated for 60 min with 100 μg/mL of NPs. Two different planes are shown, one upper plane (first row) and one lower plane closer to the coverslip (second row). The accumulation of NPs at the bottom of the coverslip increases over time. Red, Rhod-PLGA; green, albumin–FITC; blue, Hoechst. Scale bar: 20 μm.

The release kinetics were followed after incubation of neuroblastoma SH-SY5Y cells with 100 μg/mL albumin–FITC-loaded Rhod-PLGA NPs for 1, 4 and 24 h. The release of albumin–FITC from the Rhod-PLGA NPs occurs over 24 h, as indicated by the gradual decrease in the green channel intensity with respect to the red channel (and thus to a shift from yellow towards red in the overlay in Figure 6b) and quantified as the ratio between the two (Figure 6c and Supplementary Materials Figure S2). While, without washing, albumin–FITC-loaded Rhod-PLGA NPs increasingly accumulated with time at the bottom of the coverslip (Figure 6d), the amount of NPs shown in Figure 6b was dependent only on the degree of washing at the end of each incubation period. The ratio between the albumin–FITC and the Rhod-PLGA is however independent of the number of NPs present after the washes.

### 3.7. WGA Structure Is Preserved After Being Loaded into the NPs

**Figure 7:**
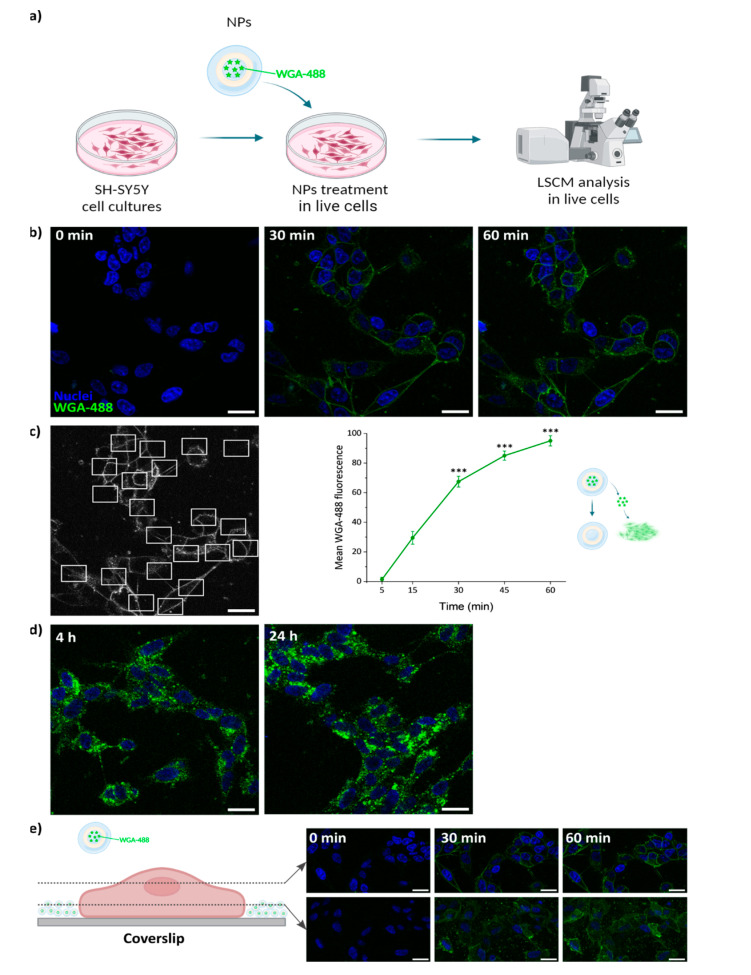
WGA-488-loaded PLGA NP release studies. (**a**) Live cell experiment workflow. Created with Biorender.com. (**b**) Representative confocal microscopy images of neuroblastoma SH-SY5Y cells incubated for 60 min with 100 μg/mL of NPs. (**c**) Representative image showing several ROIs that were selected to measure the mean fluorescence intensity of the WGA-488-stained plasma membrane, and subsequent quantification over time; error bars: SE; asterisks indicate significant differences with respect to the measurement at 5 min using Kruskal–Wallis ANOVA test followed by Dunn’s multiple comparison post hoc test (*** *p* < 0.001). (**d**) Confocal images of SH-SY5Y cells incubated for 4 and 24 h with 100 μg/mL of NPs showing the localization of WGA-488 inside the cells. (**e**) Confocal images of SH-SY5Y cells incubated for 60 min with 100 μg/mL of NPs. Two different planes are shown, one median plane (first row) and one lower plane closer to the coverslip (second row). The accumulation of NPs at the bottom of the coverslip increases over time. Green, WGA-488; blue, Hoechst. Scale bar: 20 μm.

**Figure 8:**
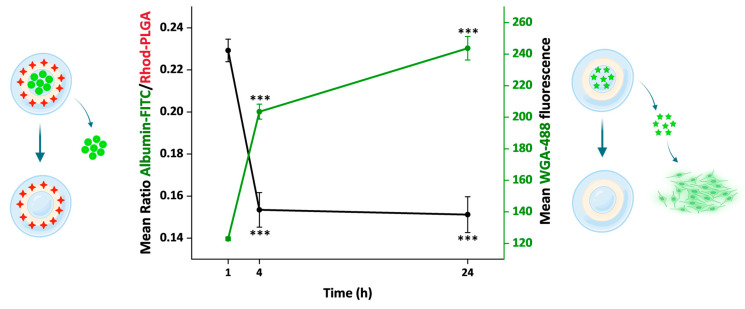
Comparison of the cargo release in 24 h. The plot shows two different measurements related to the cargo released from the NPs. On the left y axis is reported the ratio between albumin–FITC and Rhod-PLGA measured over time. On the right y axis is reported the mean fluorescence intensity values of the WGA-488 released from the NPs and staining the cell membrane. Error bars, SE; asterisks indicate significant differences with the measurement at 1 h using Kruskal–Wallis ANOVA test followed by Dunn’s multiple comparison post hoc test (*** *p* < 0.001).

To verify that the NP loading procedure did not impair the properties of the protein cargo, we verified that WGA-488 previously loaded in the PLGA NPs could correctly stain the plasma membrane after release. SH-SY5Y cells were incubated with 100 μg/mL WGA-488-loaded PLGA NPs for different incubation times. A continuous time-course experiment was performed during the first 60 min after addition of the NPs, acquiring confocal images of the same field of view every 5 min (Figure 7b). The staining of the plasma membrane following the release of WGA-488 from the NPs started to be visible as shortly as 5 min from the beginning of the incubation (Figure 6c and Supplementary Materials Video S1). A progressive increase in the mean fluorescence intensity of the cell membrane was observed over time (Figure 7c), in agreement with the release profiles observed in vitro and while incubated with cells (Figure 5 and Figure 6c). These results confirm that the structure of the loaded protein was not compromised by the encapsulation process, thus preserving its functionality. The same effect as mentioned before for the albumin-loaded NPs can be observed here. The NPs sediment and accumulate on the bottom of the coverslip over time (Figure 7e). At longer incubation times (4–24 h), internalization of the WGA-488 and accumulation in the cytoplasm were observed as a consequence of the natural membrane trafficking and remodeling processes (endocytosis, pinocytosis, etc.) occurring in the cells (Figure 7d). The total mean fluorescence intensity per cell kept increasing with time (Figure 8).

### 3.8. Release Kinetics of the Loaded Model Substances over 24 h

Overall, we used two different approaches to follow the NP cargo release, one direct and one indirect. First, using the albumin–FITC-loaded Rhod-PLGA NPs, the ratio between the albumin–FITC (cargo) and Rhod-PLGA (NPs) fluorescence intensities was measured over 24 h (Figure 6, Figure 8, and Supplementary Materials Figure S2). The ratio decreased significantly over a period of 24 h (red line, Figure 8), indicating that the amount of albumin–FITC in the NPs was gradually reduced. Second, using the WGA-488-loaded PLGA NPs, the degree of fluorescent labeling of the plasma membrane derived from the WGA-488 released from the PLGA-NPs was measured. In this case, the fluorescence intensity of the labeled membrane increased significantly over time (green line, Figure 8). Notably, the kinetic profiles obtained with the two complementary approaches appear to inversely correlate (see also Figure S3).

## 4. Conclusions

We have reported a novel continuous manufacturing method to prepare PLGA NPs. Our results showed that albumin–FITC-loaded fluorescent PLGA NPs were successfully prepared with the combination of traditional homogenization and novel continuous manufacturing methods, which offer scalability and robustness to manufacturing. The employment of a continuous manufacturing technology system in the second emulsification step of double-emulsion preparation allowed scalability in the mixing of larger volumes of phases during the emulsification. The PLGA NPs prepared by double emulsions demonstrated high encapsulation efficiency and narrow particle size distribution, also indicating the strength of the manufacturing method. The prepared particles showed sustained release properties, which was confirmed by both in vitro release studies. Moreover, the particles showed biocompatibility in two different cell lines, and they were not internalized by the cells, which is a desirable characteristic for N2B delivery. Our studies also showed that WGA488 was successfully encapsulated into the PLGA NPs by the double-emulsion method, where narrow size distribution was also achieved. We demonstrated with WGA-488-loaded PLGA NPs that the structure of the protein used as cargo is still functional after encapsulation and release of the NPs, and that the release of the cargo is sustained over time in cell culture studies. The release profile of albumin–FITC from the PLGA NPs showed similar characteristics to the release profile of the WGA-488. Overall, this study provides some insights into how this production technique can be advantageous for the development of DDSs for N2B delivery.

## Data Availability

Data are contained within the article and Supplementary Materials.

## Supplementary Materials

The following supporting information can be downloaded at https://www.mdpi.com/article/10.3390/pharmaceutics17010087/s1: Figure S1: Rhod-PLGA NP localization after 24 h of incubation. (a) Live cell experiment workflow. Created with Biorender.com. (b) Confocal scanning microscope images of neuroblastoma SH-SY5Y cells incubated for 24 h with 100 μg/mL of NPs and then with 5 μg/mL WGA-488 for 30 min to specifically label the plasma membrane. Red, Rhod-PLGA; green, WGA-488; blue, Hoechst. White arrows show the NPs’ position. Scale bar: 20 μm. Figure S2: Ratio measurement of the fluorescence between two channels, the cargo (albumin–FITC, green channel) and the NPs (Rhod-PLGA, red channel). The images are selected; a strong threshold is applied to the NP channel (561 excitation wavelength, red channel) and the particles are analyzed, delimiting the region of interest (ROI). The strong threshold assures that the fluorescence detected is coming from the NPs alone. These ROIs are selected and saved to be used in the next step. Then, applying the Ratio Plus plugin in the Fiji software, a measurement of the ratio between both channels is possible (the cargo from the green channel over the NPs from the red channel) in the ROIs previously selected that are the regions where the NPs are present. Like this, a measurement of how much cargo (albumin–FITC) is present in the NPs (Rhod-PLGA) over time is obtained. Figure S3: Correlation between the kinetics profiles. Pearson’s correlation coefficient calculated between the measurements of the ratio of cargo (albumin–FITC) and Rhod-PLGA NPs (x axis), and the fluorescence derived from WGA-488 plasma membrane staining due to the cargo release from the NPs (y axis) at 1, 4, and 24 h. The graph illustrates a strong, statistically significant inverse correlation between the two variables, confirming what is shown in Figure 8. Video S1: WGA-488-loaded PLGA NP continuous time-course experiment during the first 60 min after the addition of the NPs.
