# Supplementary material for: Scalable Manufacturing Method for Model Protein-Loaded PLGA Nanoparticles: Biocompatibility, Trafficking and Release Properties
Source: Pharmaceutics. 2025 Jan 10;17(1):87. doi: 10.3390/pharmaceutics17010087 (PMC11768205; doi:10.3390/pharmaceutics17010087)
Supplement: Supplementary file 1 [file pharmaceutics-17-00087-s001.zip › pharmaceutics-3358342-supplementary.pdf]

pharmaceutics

*Supplementary Material*

# Scalable Manufacturing Method for Model Protein-loaded PLGA Nanoparticles: Biocompatibility, Trafficking and Release Properties

**Selin Akpinar Adscheid, Marta Rojas-Rodríguez, Salma M. Abdel-Hafez, Francesco S. Pavone, Marc Schneider, Akif E. Türeli, Martino Calamai, Nazende Günday-Türeli**

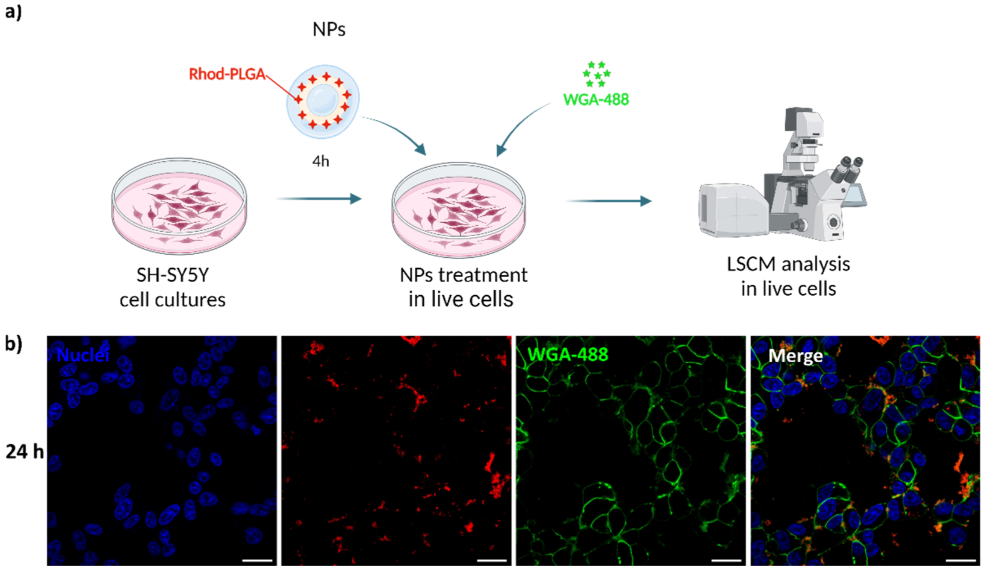


**Figure S1.** Rhod-PLGA NPs localization after 24-h of incubation. a) Live cells experiment workflow. Created with Biorender.com b) Confocal scanning microscope images of neuroblastoma SH-SY5Y cells incubated for 24 h with 100 µg/ml of NPs. Then with 5 µg/ml WGA-488 for 30 min to specifically label the plasma membrane. Red, Rhod-PLGA; green, WGA-488; blue, Hoechst. White arrows show the NPs` position. Scale bar 20 µm.

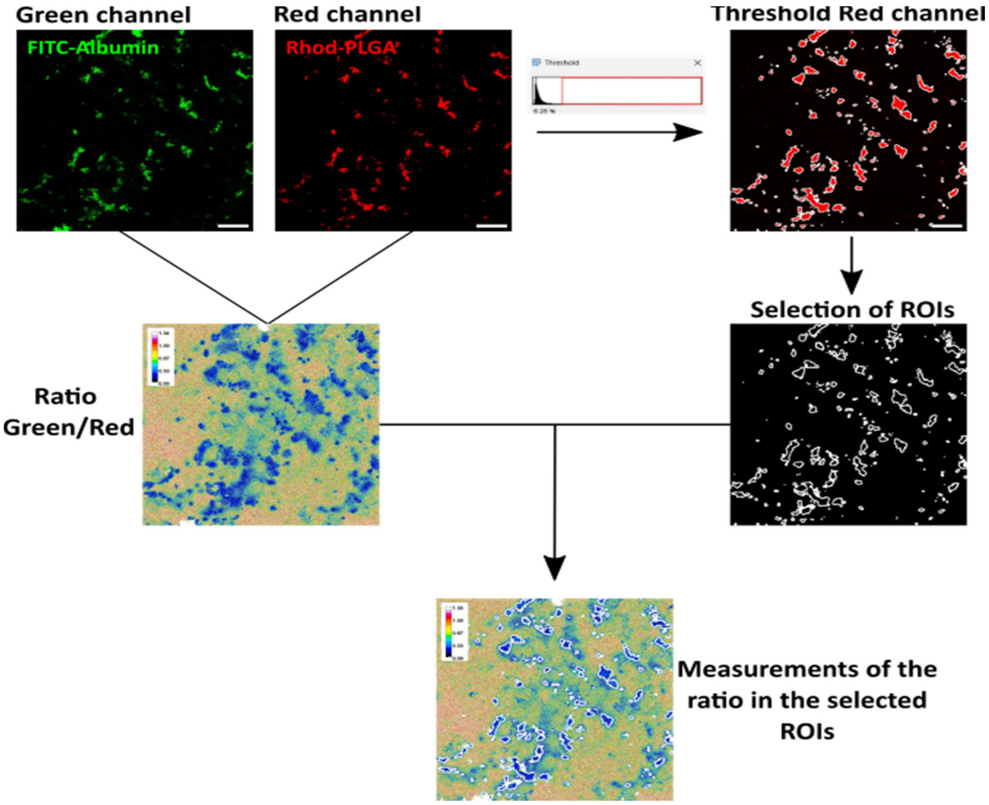


**Figure S2**. Ratio measurement

In order to measure the ratio of the fluorescence between two channels, the cargo (albumin-FITC, green channel) and the NPs (Rhod-PLGA, red channel). The images are selected, a strong threshold is applied to the NPs channel (561 excitation wavelength, red channel) and the particles analyzed delimiting the region of interest (ROI). The strong threshold assures that the fluorescence detected is coming from the NPs alone. These ROIs are selected and saved to be used in the next step. Then applying the Ratio Plus plugin in the Fiji software, a measurement of the ratio between both channels is possible (the cargo from the green channel over the NPs from the red channel) in the ROIs previously selected that are the regions where the NPs are present. Like this, a measurement of how much cargo (albumin-FITC) is present in the NPs (Rhod-PLGA) overtime is obtained.

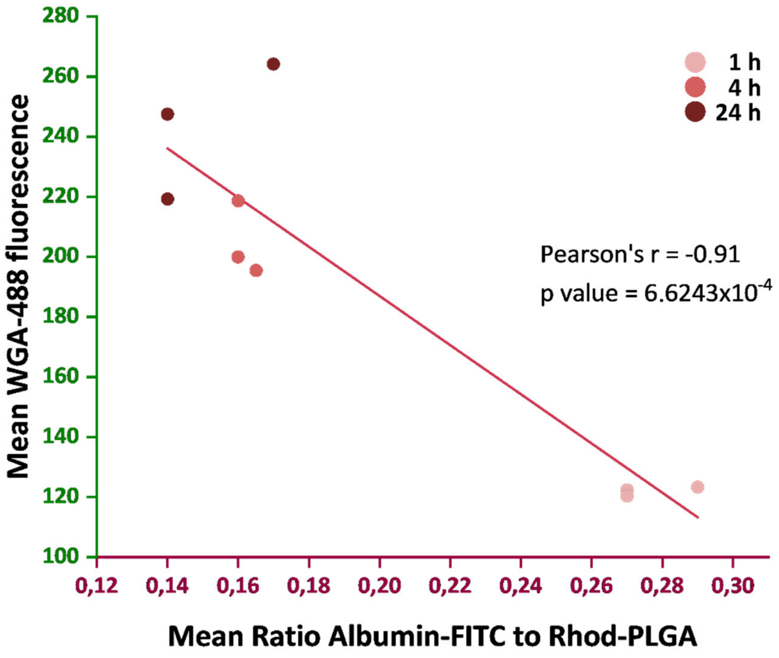


**Figure S3**. Correlation between the kinetics profiles. Pearson's correlation coefficient calculated between the measurements of the ratio cargo (albumin-FITC) Rhod-PLGA NPs (x axis), and the fluorescence derived from WGA-488 plasma membrane staining due to the cargo release from the NPs (y axis) at 1, 4 and 24 h. The graph illustrates a strong, statistically significant inverse correlation between the two variables confirming what is shown in figure 8.
