# Supplementary material for: Scalable Manufacturing Method for Model Protein-Loaded PLGA Nanoparticles: Biocompatibility, Trafficking and Release Properties
Source: Pharmaceutics. 2025 Jan 10;17(1):87. doi: 10.3390/pharmaceutics17010087 (PMC11768205; doi:10.3390/pharmaceutics17010087)
Supplement: Supplementary file 1 [file pharmaceutics-17-00087-s001.zip › Video S1.pptx]

## Slide 1
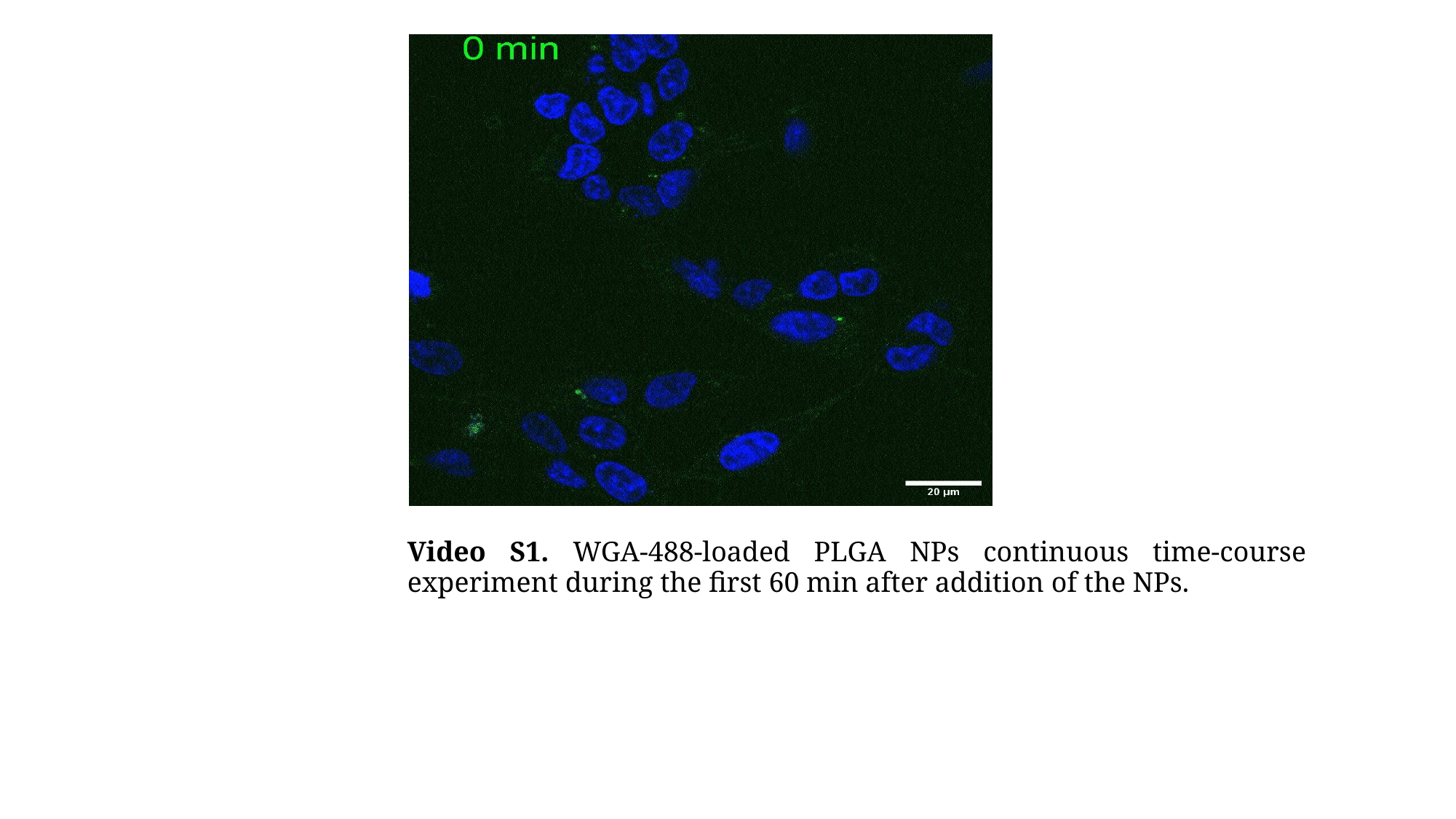

Video S1. WGA-488-loaded PLGA NPs continuous time-course experiment during the first 60 min after addition of the NPs.
